# Imaging Features of Sclerosing Epithelioid Fibrosarcoma of the Pancreas: A Case Report

**DOI:** 10.3389/fonc.2020.00901

**Published:** 2020-06-17

**Authors:** Weizhi Xia, Yunjun Yang, Yingbao Huang

**Affiliations:** ^1^Department of Radiology, The Second Affiliated Hospital of Wenzhou Medical University, Wenzhou, China; ^2^Department of Radiology, The First Affiliated Hospital of Wenzhou Medical University, Wenzhou, China

**Keywords:** pancreatic tumor, sclerosing epithelioid fibrosarcoma, computed tomography, magnetic resonance imaging, imaging features

## Abstract

**Background:** Sclerosing epithelioid fibrosarcoma (SEF) is an extremely rare fibrosarcoma variant. There is no complete imaging data on SEF involving the pancreas. Herein we report the computed tomography (CT) and magnetic resonance imaging (MRI) data of a patient with SEF that primarily involved the pancreas.

**Case Presentation:** A 64-year-old man was found to have a solid mass in the tail of the pancreas on unenhanced CT. He had no constitutional symptoms. Contrast-enhanced abdominal CT and MRI were performed, and the results of the latter provided the diagnosis of a pancreatic neuroendocrine tumor. Laparoscopic distal pancreatectomy and splenectomy were performed. Anatomopathological examination and immunohistochemistry confirmed that the tumor was a SEF of the pancreas. The patient had no signs of recurrence or metastasis during a 12-months follow-up.

**Conclusion:** We report an extremely rare case of SEF in the pancreas and its characterization with CT and MRI.

## Background

Sclerosing epithelioid fibrosarcoma (SEF) is an extremely rare variant of fibrosarcoma, first reported by Meis-Kindblom and grouped under low-grade fibrosarcoma in 1995 (1). It is characterized by fibroblastic epithelioid cells arranged in cords, nests, or sheets within a collagenous extracellular matrix (2). SEF typically involves the extremities or the torso and presents as a deep tissue mass (1–4), less commonly occurring in abdominal viscera, with only two cases involving the pancreas reported in literature to date (5). No detailed imaging report on SEF of the pancreas has been published so far. Herein we report the CT and MRI imaging data of a patient with SEF of the pancreas.

## Case Presentation

A 64-year-old man was referred to the Department of Hepatobiliary Surgery of the First Affiliated Hospital of Wenzhou Medical University due to a solid mass of the pancreas found by unenhanced CT, with no abdominal pain, bloating, nausea, vomiting, or jaundice. The patient had a prior history of hypertension for 8 years and diabetes for more than 20 years. Routine serum tumor biomarkers, including carcinoembryonic antigen (4.9 μg/L), carbohydrate antigen 125 (CA125 7.3 U/ml), carbohydrate antigen 199 (CA199 12.7 U/ml), alpha fetoprotein serum (2.31 ng/ml), and prostate-specific antigen (0.310 ng/ml), were found by chemiluminescence enzyme immunoassay to be within normal ranges.

### Imaging Examinations

**Figure 1:**
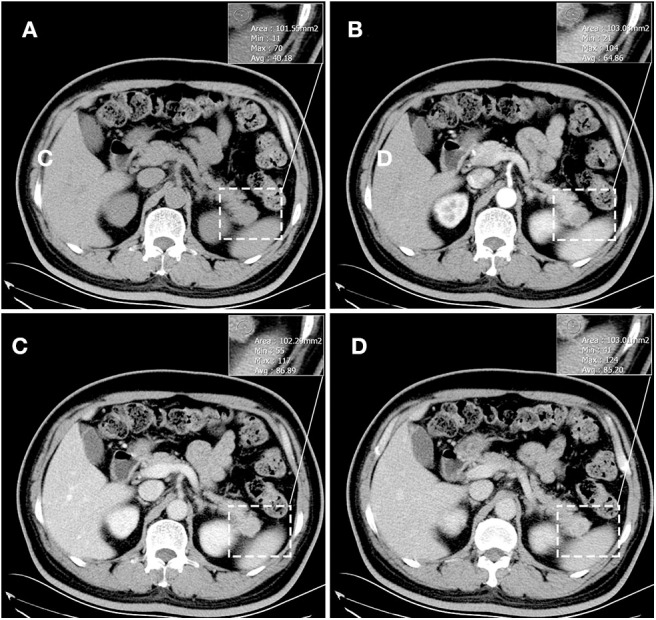
Unenhanced CT showed the mass to be isoattenuating and barely perceptible. **(A)** It demonstrated mild enhancement in the arterial phase **(B)** and moderate heterogeneous enhancement in the portal **(C)** and the late phases **(D)**. The corresponding CT values were measured (insert). There was no evidence of superior mesenteric artery or portal venous invasion.

**Figure 2:**
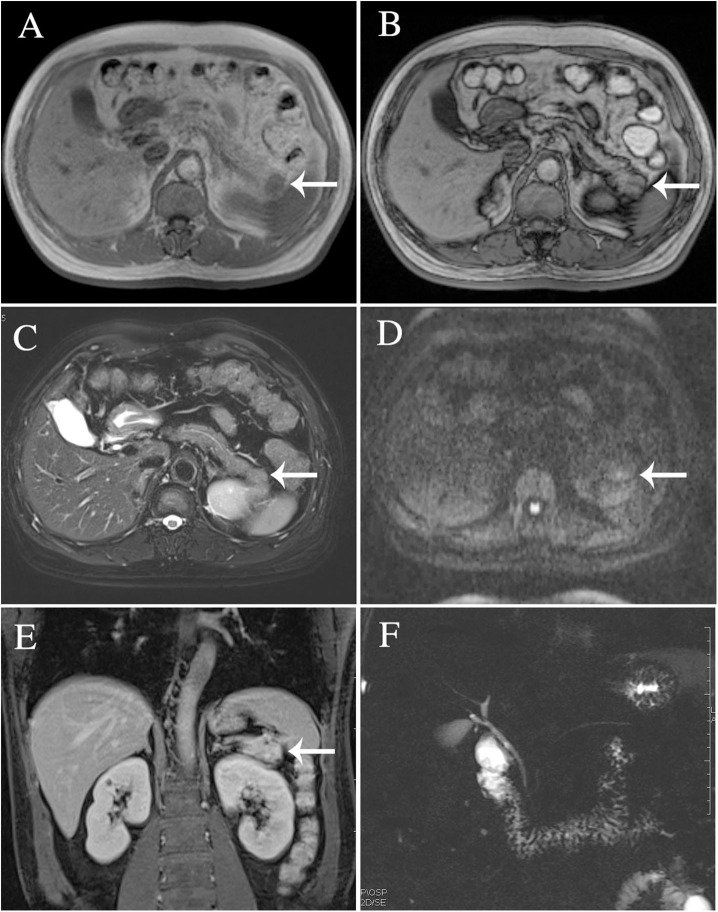
The lesion was identified in T2-weighted images **(C)**, diffusion-weighted images **(D)**, and magnetic resonance cholangiopancreatography **(F)**. There was no significant difference between the in-phase **(A)** and the out-of-phase MRI imaging **(B)**. Coronal MRI imaging **(E)**.

**Figure 3:**
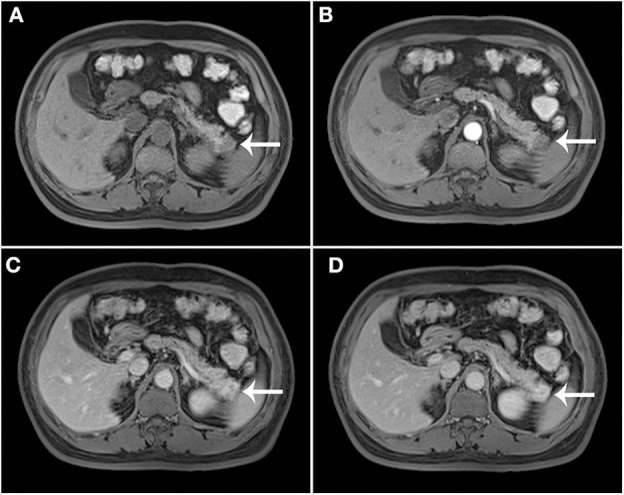
Post-contrast MRI. T1WI without contrast enhancement **(A)**, the arterial phase **(B)**, the portal **(C)**, and the late phases **(D)**.

The results of axial unenhanced CT showed the mass to be isoattenuating and barely perceptible (Figure 1A). The contrast-enhanced CT (CECT) image showed a well-defined regular nodular mass (2.0 × 1.5 × 1.8 cm) in the pancreatic tail. It demonstrated mild enhancement in the arterial phase (Figure 1B) and moderate heterogeneous enhancement in the portal (Figure 1C) and the late phases (Figure 1D). There was no evidence of superior mesenteric artery or portal venous invasion. On unenhanced MRI, the lesion was hypointense on T1-weighted images (T1WI) (Figure 3A), hyperintense on T2-weighted images (T2WI) (Figure 2C), and slightly hyperintense on diffusion-weighted images (DWI) (Figure 2D). There was no significant difference between the in-phase (Figure 2A) and the out-of-phase MRI (Figure 2B) and no signs of necrosis, hemorrhage, or cyst formation. The coronal MRI image also showed the mass in the pancreatic tail (Figure 2E). The magnetic resonance cholangiopancreatography (MRCP) images showed no expansion of the pancreatic duct, extrahepatic bile duct, or intrahepatic duct (Figure 2F). After contrast agent (Gd-DTPA) administration (Figures 3B–D), the dynamic enhancement pattern was similar to that of CECT. The MRI report suggested a pancreatic neuroendocrine tumor (PNET). The results of abdominal CT and MRI showed no signs of metastasis.

### Surgical Findings and Anatomopathological Examination Results

**Figure 4:**
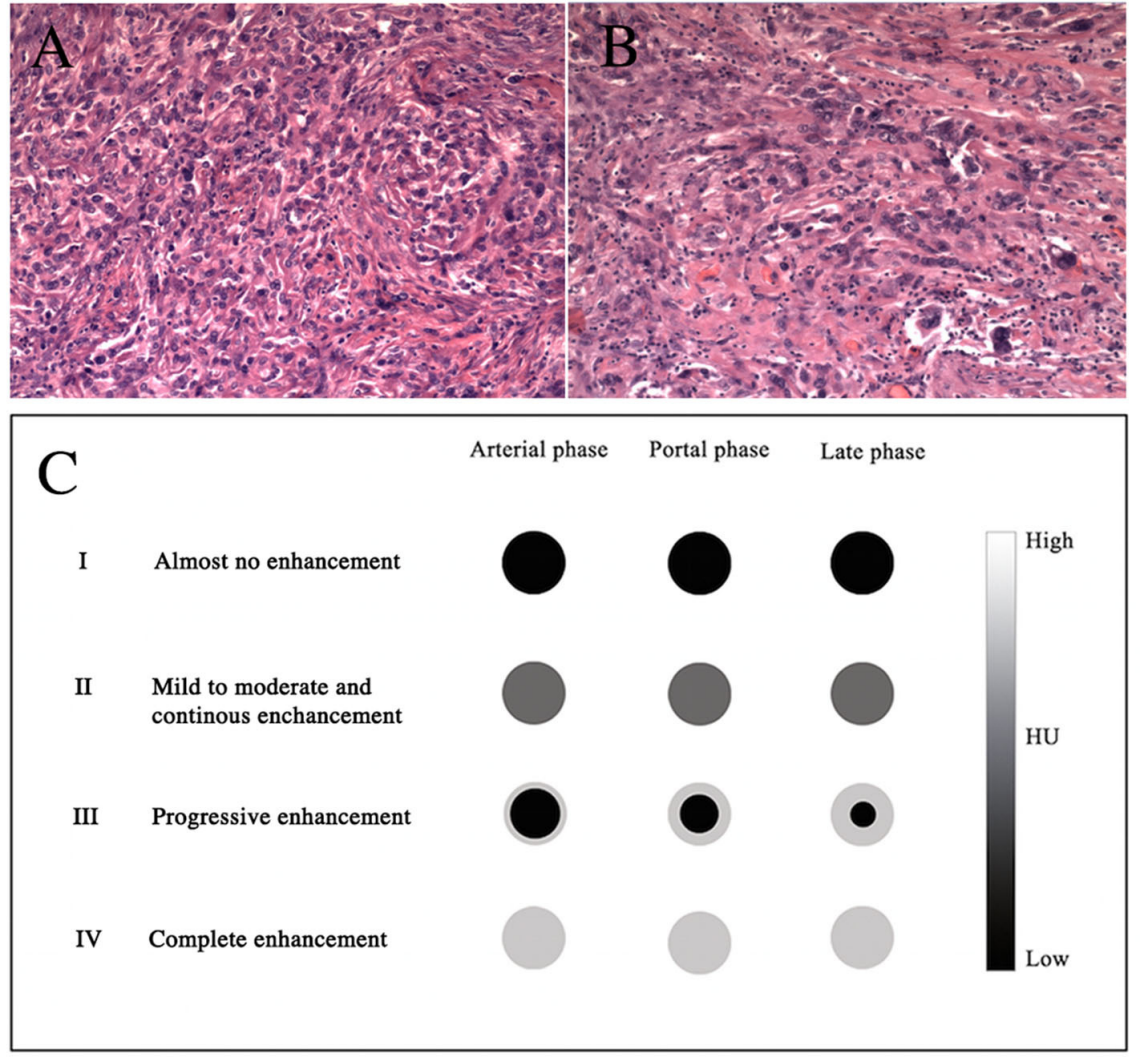
H&E stains **(A,B)**, original magnifications ×100 and ×200, respectively. The diagram shows the dynamic enhancement patterns of the common tumors of the pancreas **(C)**.

Based on these findings, on March 29, 2018, laparoscopic distal pancreatectomy and splenectomy were performed. The tumor was well-encapsulated, located in the tail of the pancreas, and close to the spleen. The size of the tumor was 1.5 cm in maximum diameter. There were no ascites and no visible metastatic nodules found in the pelvis, liver, or omentum. Upon histological staining, it was revealed that the fibroblastic epithelioid cells were ovoid and arranged in nests (Figure 4A), cords, or sheets (Figure 4B) within a collagen-rich extracellular matrix. Immunohistochemical staining was performed to further characterize this lesion. The tumor cells were positive for vimentin, BcL-2, CD99, epithelial membrane antigen (EMA), and smooth muscle actin (SMA) and were negative for CD34, CD56, CgA, S-100(–), and Syn(–). The Ki-67 proliferation index was low (8%+) in tumor cells. A diagnosis of SEF was made.

### Post-operative Course

After 12 months of follow-up, the patient had no signs of recurrence or metastasis.

## Discussion

SEF is an extremely rare fibrosarcoma variant that is grouped under low-grade fibrosarcomas (1). SEF mainly affects patients with a mean age of 47 years and has no sex predilection (4). Our patient was a 64-year-old man. It typically arises in the extremities or the torso (1–4) and less commonly in the abdominal viscera, with only two cases involving the pancreas reported in literature to date (5).

SEF is histologically characterized by fibroblastic epithelioid cells arranged in cords, nests, or sheets within a collagenous extracellular matrix (2, 5). The histological differential diagnosis of SEF is extremely difficult. Immunohistochemistry is vital for making definite diagnoses. It typically includes the determination of cytokeratins, EMA, common leukocyte antigen, SMA, protein S-100, neurospecific enolase, desmin, and vimentin (6–10). In most SEF cases, there is a consistent and strong expression of vimentin (1). In our case, the tumor cells were positive for SMA, EMA, CD99, BcL-2, and vimentin.

The rapid development of clinical imaging has been shown to be of great value in pre-operative oncological diagnosis. No detailed imaging report on SEF of the pancreas has been published so far. Our case is the first in which the patient has both CT and MRI imaging data. The image analyses provide information regarding the size, location (head/body/tail), shape (round/oval/lobulated), margins (regular/irregular), density on CT (hypo-, iso-, or hyperattenuating and homogeneous or heterogeneous) or signal intensity on MRI (hypo-, iso-, or hyperintense), and the dynamic enhancement pattern as well as the presence or absence of necrosis, hemorrhage, or cyst formation. Furthermore, it can reveal the presence or absence of vascular invasion, pancreatic or bile duct expansion, lymph node metastasis, or distant metastasis. The dynamic enhancement patterns of common tumors of the pancreas are as follows (Figure 4C): type I, almost no enhancement; type II, mild to moderate and continuous enhancement; type III, progressively increasing centripetal enhancement toward the center of the lesion; and type IV, complete and immediate enhancement. The tumor in our case appeared as isoattenuating (CT), T1 hypointense, T2 hyperintense, DWI hyperintense (MRI), well-defined, and round. The dynamic enhancement pattern of SEF was mild on the arterial phase and moderately heterogeneous on the portal and the late phases. This relatively delayed and prolonged enhancement suggested type II. There were no signs of vascular invasion, pancreatic or bile duct expansion, or metastases. A pathological analysis revealed that SEF was a distinctive variant of fibrosarcoma characterized by abundant fibroblastic epithelioid cells arranged in cords, nests, or sheets within a collagenous extracellular matrix (2, 5). The hypercellular tumor showed as isointense or hypointense on T1WI, hyperintense or isointense on T2WI, and hyperintense on DWI due to the limited motion of water protons (11). The relatively delayed enhancement areas may be correlated with collagen tissue, and the prolonged enhancement areas may be correlated with the abundant hypercellular areas (12, 13). In addition, we hypothesized that heterogeneous enhancement might have resulted from non-uniform cell arrangement or fibrous components. Imaging is broadly used for the differential diagnosis of SEF. Pancreatic ductal adenocarcinoma accounts for 80–90% of all pancreatic tumors, in which the most significant imaging feature is hypoenhancement. It shows almost no enhancement against the background of enhancing pancreas (14), which suggests that it is type I. PNET makes up 2–10% of all pancreatic tumors, and typically appears as a well-circumscribed hypervascular solid mass with avid enhancement on the pancreatic arterial and portal venous phase (15). The dynamic enhancement pattern of PNET suggests that it is type IV. However, approximately 41.5% of PNETs may not show hypervascular appearance but either iso- (type II) or hypo-enhancement (type I) in the arterial phase (15–17). In addition, solid pseudopapillary neoplasm (SPN) of the pancreas comprises 0.9–2.7% of all pancreatic tumors. The typical imaging appearance of SPN has been described as a large mass with heterogeneous solid and cystic areas or purely solid and with progressively increasing centripetal enhancement toward the center of the lesion (18), which is consistent with type III.

The treatment regimen for SEF has not been fully established. Surgical resection of localized tumors remains the most effective treatment for SEF (5). The effectiveness of systemic adjuvant therapy to improve the progression of SEF remains unclear (3, 4). Owing to its aggressive behavior, the prognosis of SEF is typically poor. After 12 months of follow-up, our patient had no signs of recurrence or metastasis, and imaging follow-up is still needed.

## Conclusion

We present an extremely rare case of SEF in the pancreas and its characterization with CT and MRI. Imaging is highly valuable for pre-operative oncological diagnosis.

## Data Availability Statement

All datasets generated for this study are included in the article/supplementary material.

## Ethics Statement

The studies involving human participants were reviewed and approved by The First Affiliated Hospital of Wenzhou Medical University Biomedical Research Ethics Committee. The patients/participants provided their written informed consent to participate in this study. Written informed consent was obtained from the individual(s) for the publication of any potentially identifiable images or data included in this article.

## Author Contributions

WX performed the data acquisition. WX and YH performed the data analysis and interpretation. YY performed the radiological analysis of MRI and CT images. YH and YY performed the manuscript preparation. All authors contributed to the article and approved the submitted version.

## Conflict of Interest

The authors declare that the research was conducted in the absence of any commercial or financial relationships that could be construed as a potential conflict of interest.
